# CEACAMS 1, 5, and 6 in disease and cancer: interactions with pathogens

**DOI:** 10.18632/genesandcancer.230

**Published:** 2023-02-01

**Authors:** Jerin Thomas, Addison Klebanov, Sahara John, Larry S. Miller, Anil Vegesna, Richard L. Amdur, Krishanu Bhowmick, Lopa Mishra

**Affiliations:** ^1^Donald and Barbara Zucker School of Medicine, Hempstead, NY 11549, USA; ^2^The Institute for Bioelectronic Medicine, The Feinstein Institutes for Medical Research, Northwell Health, Manhassett, NY 11030, USA; ^3^Department of Medicine, The Institute for Bioelectronic Medicine, The Feinstein Institutes for Medical Research, Division of Gastroenterology, Northwell Health, Manhassett, NY 11030, USA; ^4^Quantitative Intelligence Unit, The Institutes for Health Systems Science and Bioelectronic Medicine, The Feinstein Institutes for Medical Research, Northwell Health, Manhassett, NY 11030, USA; ^5^Cold Spring Harbor Laboratory, Cold Spring Harbor, NY 11724, USA

**Keywords:** CEACAMs, cancer, immunology, inflammatory diseases

## Abstract

The CEA family comprises 18 genes and 11 pseudogenes located at chromosome 19q13.2 and is divided into two main groups: cell surface anchored CEA-related cell adhesion molecules (CEACAMs) and the secreted pregnancy-specific glycoproteins (PSGs). CEACAMs are highly glycosylated cell surface anchored, intracellular, and intercellular signaling molecules with diverse functions, from cell differentiation and transformation to modulating immune responses associated with infection, inflammation, and cancer. In this review, we explore current knowledge surrounding CEACAM1, CEACAM5, and CEACAM6, highlight their pathological significance in the areas of cancer biology, immunology, and inflammatory disease, and describe the utility of murine models in exploring questions related to these proteins.

## INTRODUCTION

Carcinoembryonic antigen (CEA), one of few FDA-approved biomarkers for cancer, was first identified and described in 1965 as a tumor-specific antigen expressed in embryonic gut, liver, and pancreas tissues, as well as gastrointestinal and respiratory malignancies, but not in differentiated adult tissues [1]. Like other developmental pathways and members, such as WNT, NOTCH, and Hedgehog, involved in many aspects of embryogenesis, CEA is repressed in the normal human digestive and respiratory systems but reappears during malignant transformation [1, 2]. Discovery of similar CEA-like proteins led to a nomenclature meeting reorganizing the family of proteins as carcinoembryonic antigen-related cellular adhesion molecules, or CEACAMs [3]. Notably, CEACAM1, CEACAM5, and CEACAM6 have been found to be implicated in immune related disease and cancer [4–8], and are now considered valid clinical biomarkers and promising therapeutic targets in melanoma, lung, colorectal, and pancreatic cancers. CEACAM1 is a biomarker in melanoma [9, 10], non-small cell lung cancer [11] and pancreatic adenocarcinoma (PDAC), and its increased expression is associated with severe disease. CEACAM5 has a significant clinical role as a tumor marker for several tumors including gastrointestinal and respiratory malignancies [6, 12, 13]. However, due to low sensitivity and specificity, its predictive value alone is still unclear. CEACAM6 is highly expressed in hyperplastic polyps and colon adenomas [14], breast cancer, pancreatic cancer, mucinous ovarian cancer, gastric cancer, and lung adenocarcinoma [15].

**Figure 1:**
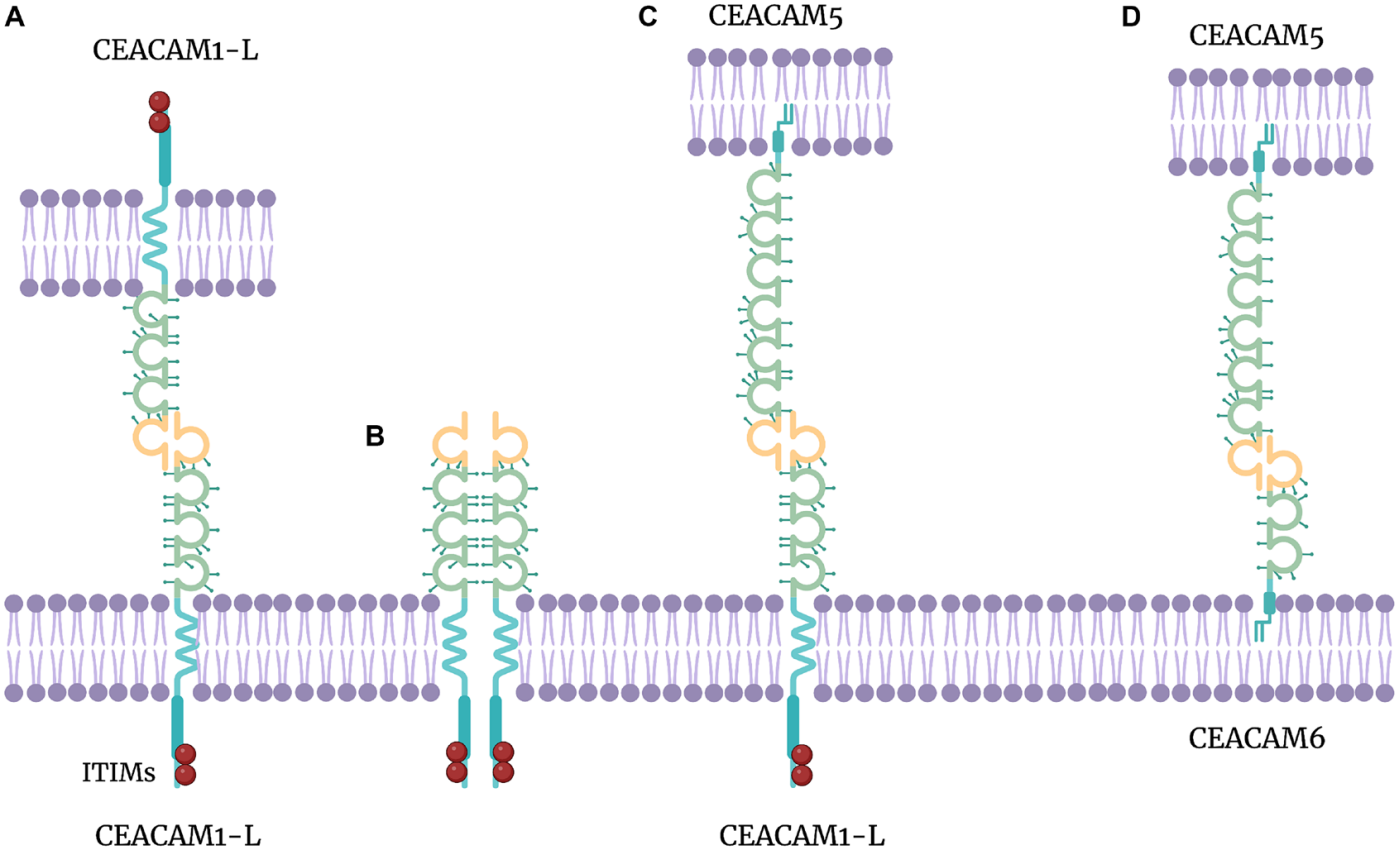
CEACAM interactions. (**A**) CEACAM *trans*-homophilic binding (CEACAM1-L – CEACAM1-L). (**B**) CEACAM *cis*-homodimer (CEACAM1-L – CEACAM1-L). (**C**) CEACAM *trans*-heterophilic binding (CEACAM5 – CEACAM1). (**D**) CEACAM heterophilic binding (CEACAM5 – CEACAM1).

All CEACAMs contain an N-terminal V set fold from the immunoglobulin (Ig) superfamily, up to three type 2 immunoglobulins, a transmembrane domain, and a cytoplasmic domain, which facilitate adhesion through homophilic (CEACAM1, CEACAM5, and CEACAM6) and/or heterophilic (CEACAM1–CEACAM5, CEACAM5–CEACAM6, and CEACAM6–CEACAM8) interactions (Figure 1) [4]. Each CEACAM has a specific expression pattern [14, 16, 17]. Depending on the cell type, CEACAM1, CEACAM5, and CEACAM6 play pivotal roles in particular aspects of cancer biology and immunology [14, 18, 19].

Modeling the function of CEACAMs on tumor growth, the tumor microenvironment, the immune system, and inflammatory disease has been particularly challenging, with mouse models for only a tiny number, reflecting limited expression in rodents: CEACAM1 (knockout), and collectively CEABAC transgenic pooling CEACAM3, CEACAM5, and CEACAM6 [20]. Despite this, transgenic mouse and knockout models provide substantial insight into the role of CEACAMs in disease [20–22].

## FUNCTION AND EXPRESSION PATTERNS OF CEACAMS 1, 5, AND 6

CEACAM1 expression is the broadest, found in the cytoplasm of granulocytes and myeloid cells, and in the endothelium of the thyroid gland, adrenal gland, endometrium, prostate, and placenta [17]. Moreover, CEACAM1 is expressed on the cell membrane of esophageal glands, uterine glands, pancreatic ducts, prostate and cervix epithelia, liver bile ducts, enterocytes, hepatocytes, and goblet cells, with particularly strong expression in colonic absorptive cells and renal epithelia [17]. CEACAM5 is expressed similarly in the colon and pancreas but is expressed on the cell surface of gastric and intestinal mucous cells, with particularly strong staining in esophageal squamous epithelia [17]. CEACAM6 is abundantly expressed in the colon, liver, stomach, gallbladder, skin, and tongue [17]. In addition, it is expressed in the squamous epithelia of the tongue, esophagus and cervix. CEACAM6 is also prominent in the mucous epithelia of the submandibular salivary and the anterior lingual gland, as well as myeloid cells in the prostate gland and bone marrow [14].

CEACAM1 comprises several isoforms through alternative splicing, yet the functions of each of these isoforms remain to be fully elucidated. CEACAM1 isoforms differ in the amount of extracellular immunoglobulin-like domains and the length of the cytoplasmic tail. The short tail isoform (CEACAM1-S) does not have any immunoreceptor tyrosine-based inhibitory motifs (ITIMs), but contains sequences that can bind to calmodulin [23], tropomyosin, and F-actin [24]. The interaction between calmodulin and CEACAM1 results in the interference of CEACAM1 *cis*-homodimerization, suggesting that calmodulin regulates the activity of CEACAM1 [23]. In addition, co-sedimentation assays reveal that a glutathione S-transferase fusion protein containing the S-isoform fusion protein (GST-Cyto-S) binds to F-actin and tropomyosin, especially when incubated with G-actin during polymerization [24]. The long tail variant (CEACAM1-L) has two ITIMs that negatively regulate signaling from various activating receptors, including the T cell antigen receptor (TCR) [25]. Notably, the ratio of CEACAM1-L to CEACAM1-S varies by cell type, activation state, and growth phase [4, 26, 27].

CEACAM1 has several binding targets: intracellularly, it forms *cis*-dimers, which are essential in cytoplasmic signaling. In B cells, the dimeric state of CEACAM1 regulates recruitment of signaling molecules, such as the Src-family kinases and the Src homology 2 (SH2)-domain-containing protein tyrosine phosphatases (SHP1 & 2), by the cytoplasmic tail to the B cell receptor [28]. Upon phosphorylation, ITIMs on the CEACAM1-L isoform cytoplasmic tail bind to SHP1 and SHP2 and attach to B cell receptors (BCR), inhibiting BCR-induced Ca^2+^ mobilization [28]. CEACAM1 initially undergoes *trans*-homophilic (CEACAM1 - CEACAM1), or *trans*-heterophilic (CEACAM - CEA Family Member) dimerization, and can bind to other proteins and microbes [4, 29]. For example, *trans*-homophilic dimerization of CEACAM1 can protect monocytes from apoptosis through the activation of the Bcl2 protein via the phosphatidylinositol 3-kinase (PI3K)/Akt pathway [30]. CEACAM1 is involved in a variety of processes, including angiogenesis [31, 32], metabolic regulation [33], and immune modulation [27, 34–41]. In angiogenesis, CEACAM1 stimulates the proliferation, chemotaxis, and capillary-like tube formation of human microvascular endothelial cells [31], prompting further exploration into the angiogenic properties of CEACAM1 as treatment towards vascular diseases, such as atherosclerosis. Furthermore, CEACAM1 is expressed in the capillaries of numerous solid human tumors such as bladder and prostate cancer, leydig cell tumors, seminomas, and brain hemangioblastoma [31].

In immune modulation, CEACAM1 is strongly upregulated in T cells following activation by cytokines. After mitogen stimulation, CEACAM1 is rapidly mobilized in T cells from an intracellular compartment to the cell surface [42]. Based on its kinetics in T cells and inhibitory role in B cells, CEACAM1 likely plays an inhibitory role in T cell immune function. In metabolic regulation, deletion or inactivation of CEACAM1 impairs insulin clearance in mice, compromising metabolic homeostasis and promoting obesity, hepatic steatosis, and fibrosis [33]. Its signaling is complex and depends on the type and stage of tissues involved, with growth-suppressive roles in certain types of tissue but proliferative and stimulative roles in other tissue types [18]. For example, reinsertion of CEACAM1 isoforms in colorectal and prostate CEACAM1-negative tumor cells inhibits xenograft tumor development in syngeneic mice, suggesting that CEACAM1-L behaves as a tumor suppressor protein; on the contrary, CEACAM1-L overexpression in tumors correlates with metastatic spread in other types of aggressive cancers, such as hepatocellular cancer [43], melanoma, non-small cell lung, gastric, thyroid, and bladder cancers [19].

**Figure 2:**
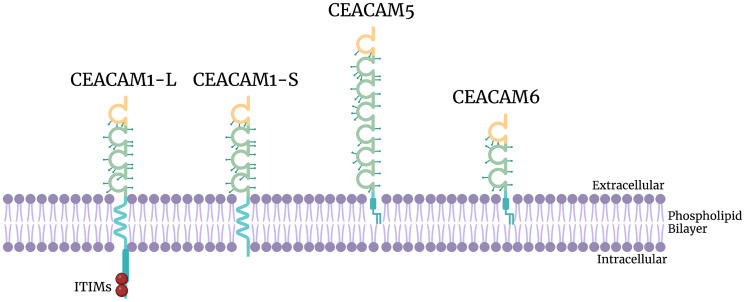
Structure of CEACAM1-L, CEACAM1-S, CEACAM5, and CEACAM6. CEACAM1-L isoform, CEACAM1-S isoform, CEACAM5, and CEACAM6. CEACAM1 is a transmembrane protein while CEACAM5 and CEACAM6 are GPI linked to the external surface. Yellow represents N-terminal variable domains; green represents C2-like Ig domains; blue represents GPI linkers (CEACAM5/CEACAM6) or transmembrane and cytosolic portions of the CEACAM (CEACAM1); red circles represent ITIM motifs; spikes on the extracellular domain represent glycosylation sites of CEACAMs.

CEACAM5 (also known as CEA) comprises one N-terminal variable domain and six C2-like Ig domains [44]; its glycosylphosphatidylinositol (GPI) linker provides membrane anchoring (Figure 2) [19, 45]. CEACAM5 normally functions as an adhesion molecule [46], but it also has roles in regulating differentiation [47], immune modulation [48, 49], and inhibiting anoikis [50]. In the rat L6 myoblast cell line (a well-characterized differentiating system), stable CEA overexpression leads to fusion into myotubes, forming multinucleated myotubes. Moreover, the entire molecular program of differentiation, including creatine phosphokinase upregulation, myogenin upregulation, and β-actin downregulation, is completely abrogated by the ectopic expression of CEACAM5, suggesting that the expression of CEACAM5 inhibits terminal differentiation [47]. Furthermore, L6 rat myoblast cells transfected with CEACAM5 and CEACAM6 undergo significantly less anoikis than L6 myoblasts expressing CEACAM1 [50]. CEACAM5 is an FDA-approved diagnostic tumor marker for colon cancer, with potential as a prognostic marker [51].

CEACAM6 has an N-terminal variable domain followed by two C2-like Ig domains and is also linked to the membrane by a GPI linker (Figure 2) [45]. Like CEACAM5, CEACAM6 was shown to be a regulator of anoikis through a mechanism involving Src and focal adhesion kinase (FAK) [52]. CEACAM6 activates the Src-FAK signaling system in a dose-dependent manner, which leads to phosphorylation of MAPK/extracellular signal-regulated kinase 1/2 (MEK1/2) and extracellular signal-regulated kinase (ERK). However, there was no observed effect on Src-FAK signaling in cells treated in the same manner with CEACAM5, suggesting that CEACAM6, not CEACAM5, forms *trans*-dimers and activates the Src-FAK pathway. Anoikis is significantly reduced in lung cancer cells with increased expression of CEACAM6, indicating that CEACAM6 plays an essential role in inhibiting anoikis through the activation of the Src-FAK signaling system [52].

## INFLAMMATORY BOWEL DISEASE

The CEACAM family of genes plays a significant role in immune modulation. A key area of study is the role of CEACAMs in binding to pathogens and their importance in inflammation. CEACAM1 is a member of the CEACAM family that is expressed in immune cells and can directly regulate immune cell activity [4, 53]. It is the only family member expressed on activated T cells [18], and it is involved in inflammatory bowel disease (IBD). Decreased expression levels of CEACAM1 are observed in pediatric Crohn’s disease (CD); however, this decrease is not found in ulcerative colitis (UC) or adult patients [7, 54]. Inhibition of CEACAM1 leads to a hyperinflammatory state that induces progression and worsens the presentation of colitis in murine models, nullifying the normal critical immunosuppressive effect of CEACAM1 [36, 39, 41, 55]. CEACAM1 expression also decreases intestinal permeability and increases epithelial barrier function in a murine model of colitis induced by dextran sulfate sodium (DSS), decreasing severity and symptomatology of the disease [56]. The increased expression of CEACAM1 in this model leads to decreased levels of proinflammatory cytokines such as TNFα and IL-6 and increased expression of tight junction proteins such as claudin1 and occludin [56]. The data suggest a protective role of CEACAM1 in models of colitis by modulating tissue and the immune environment.

CEACAM5 expression is increased in adult UC, decreased in pediatric patients, but not altered in adult CD [7]. CEACAM5 is involved in the activation of CD8^+^ suppressor T cells [57]. Normally, intestinal epithelial cells can promote CD8^+^ suppressor T cell activation by presenting soluble antigens to T cells, limiting inflammation. In patients with IBD, intestinal epithelial cells are unable to activate CD8^+^ suppressor T cells, leading to a proinflammatory state mediated by dysregulated cytokine release by CD4^+^ Th cells [58]. CEACAM6 expression is increased in pediatric CD and adult UC and CD. Compared to the role of its family members in IBD, CEACAM6 is more known for its function as a receptor for adherent invasive *E. coli* (AIEC) that contribute to the inflammation observed in IBD [59]. CEACAM6 expression is increased in a proinflammatory environment, and binding to AIEC LF82 would allow for continued inflammation and an increase in the availability of receptors that permit AIEC LF82 colonization, invasion, and the potential to alter the microbiome as this bacteria outcompetes other species [59, 60]. However, it is to be noted that other strains of AIEC do not induce the CEACAM6 expression [7, 59].

## INTERACTIONS WITH PATHOGENS

CEACAM1 is expressed on T cells following activation, and binding to extracellular ligands or pathogens can lead to T cell downregulation [42]. Several pathogens bind to CEACAMs, including several species of *Neisseria* [61, 62], *Haemophilus influenzae* [62, 63], *Helicobacter pylori* [64], *Moraxella catarrhalis* [65], *Fusobacterium nucleatum* [66], *Escherichia coli* [59, 62, 67, 68], and species of *Salmonella* [68]. A mechanism of immune suppression by *Neisseria* involves binding their opacity-associated proteins (Opa) to CEACAM1 on immune cells and inhibiting their function without being phagocytosed [38].

**Figure 3:**
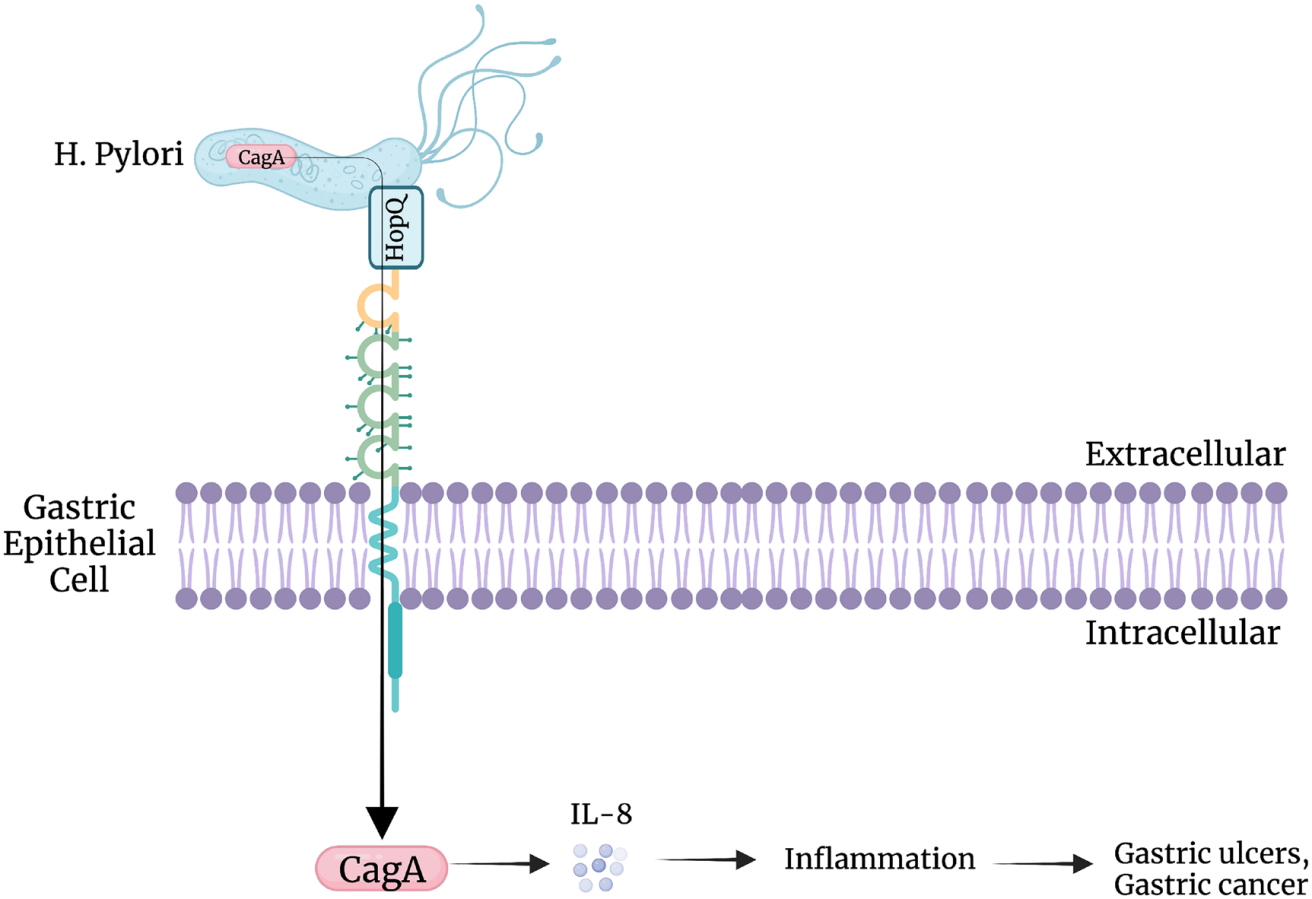
CEACAM-HopQ interaction. HopQ, the surface exposed adhesin of *H. pylori*, binds to CEACAM1, allowing for the secretion of virulent factor,CagA. This secretion enhances the release of inflammatory mediators, such as interleukin 8, ultimately resulting in gastric ulcers and gastric cancer.

Potentially, other pathogens binding to CEACAMs exhibit similar effects [38]. *Helicobactor pylori* express HopQ (a surface exposed adhesin), which allows it to bind to CEACAMs, including CEACAM1, 3, 5 and 6 [69]. This binding allows an avenue of insertion for the CagA virulence factor into cells which then increases the ability of this bacteria to colonize gastric tissue and exacerbate pathologies associated with *Helicobactor pylori* (Figure 3) [64, 69]. *H. pylori* exploit human CEACAMs, using them to gain access to the cell and inject CagA into gastric epithelial cells via bacterial type IV secretion system (T4SS) [70]. After injection, CagA promotes neoplastic transformation by interfering with intracellular signaling. CagA phosphorylation and translocation into host cells is observed in CEACAM-humanized murine neutrophils, but not WT mouse neutrophils, suggesting a CEACAM driven role in CagA activation, phosphorylation, and neoplastic transformation [71]. *H. pylori* was shown not to interact with non-human CEACAMs, including mice, and strains of *H. pylori* that could colonize the gastric mucosa of mice lack a functional T4SS and do not enumerate the pathology observed in humans [72, 73]. CEACAM-humanized neutrophils and macrophages infected by CagA-positive strain of *H. pylori* significantly enhance MIP-1α production, leading to a large influx of active inflammatory cells in the lamina propria of gastric mucosa [71]. Infection with *H. pylori* is a risk factor for developing gastric ulcers and gastric cancer (Figure 3) [74]. The *H. pylori* cytotoxin-associated gene (CagA) is the most potent risk factor for gastric cancer [70, 74]. Blocking *H. pylori* from binding CEACAM1 may prevent CagA from disrupting cell signaling and inhibiting gastric ulcers and gastric cancer.

*Streptococcus agalactiae*, also known as group B *Streptococcus* (GBS) expresses β-IgI3, a newly discovered immunoglobulin fold that binds to CEACAM1 through its small α-helix and loop. In contrast, HopQ uses an intrinsically disordered loop that folds into a β hairpin and a small helix upon binding to CEACAM1 [75]. Although they bind to CEACAM1 in a structurally different manner, β-Ig13 and HopQ target the same set of CEACAM1 residues [75]. Blocking those shared residues might prove critical in preventing bacterial accumulation. Novel β-IgI3, or similar sequences, was identified in 296 proteins expressed by human gram-positive bacteria and pathogens, including *S. oralis, S. pyogenes* (also known as group A *Streptococcus*), *S. dysgalactiae* and *S. mitis, S. intermedius, S. pneumoniae, S. pseudopneumoniae* and *Gemella haemolysans* [75]. Exploring the role of the bacterial protein β-IgI3 in adhesion to host epithelium through binding to CEACAM1 may provide further insight into the role of CEACAMs as a docking target for both commensal and pathogenic bacteria.

Following enterotoxigenic *Escherichia coli* releasing heat-labile toxin in the colon, increased cyclic AMP levels are released, ultimately leading to increased CEACAM6 expression, which in turn binds to the pathogen [67]. Therefore, increased CEACAM6 may be causal in the subsequent enteropathy associated with infection of this pathogen [67]. Although immune suppression and adhesion are beneficial in some contexts, these pathogens have found ways to exploit these proteins to create a suitable environment for infection and immune evasion. Thus, disrupting the interactions between pathogens and CEACAMs is an essential avenue of interest and one that is currently being investigated [64, 74].

## CANCER

**Figure 4:**
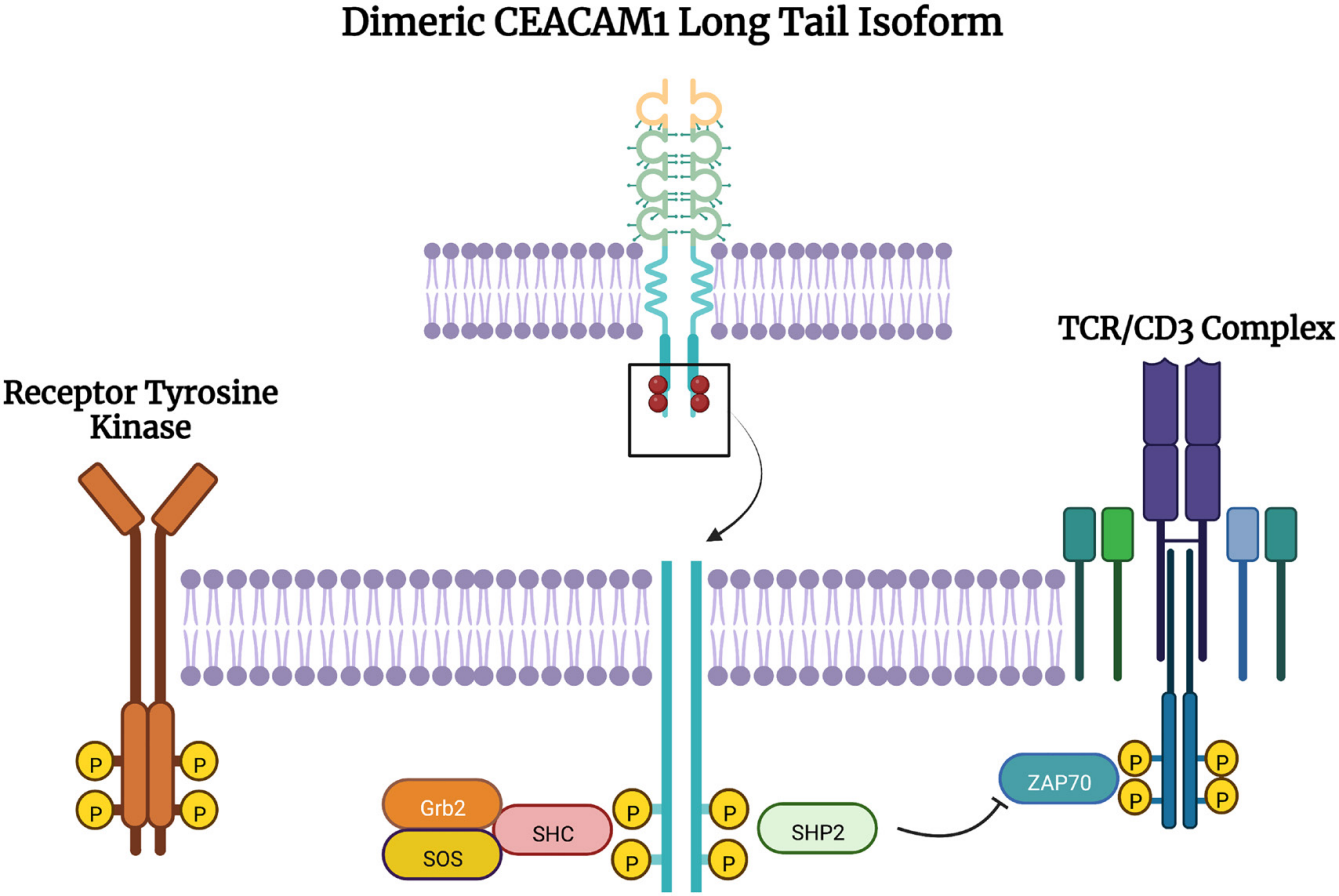
Signaling of dimeric CEACAM1 long tail isoform in epithelial cells and T cells. In epithelial cells, CEACAM1 is phosphorylated at the ITIM residues, namely Tyr493, leading to recruitment of Shc, sequestering Grb2 and SOS, reducing signaling downstream of a receptor tyrosine kinase such as insulin receptor or epidermal growth factor receptor. In T cells, dimeric CEACAM1 is phosphorylated at the ITIM residues leading to the recruitment of SHP2, whose phosphatase action reduces signaling downstream of the TCR/CD3 complex.

CEACAM1, CEACAM5, and CEACAM6 all have variable roles in tumor initiation, progression, and metastasis. The dual roles of CEACAM1 can be best understood through the separate functions of the extended versus short tail. ITIMs on CEACAM1-L bind to other extracellular ligands, including other CEACAM family members and CEACAM1, suppressing immune cells and allowing immune evasion by cancer cells [40, 75–78]. The cytoplasmic tail of CEACAM1 has several residues that serve as binding sites or phosphorylation sites for a variety of proteins that include: the Src family of kinases, insulin receptor, epidermal growth factor receptor upon EGF treatment, SHP1, SHP2, phorbol ester inducible staurosporine-inhibitable Ser/Thr kinases, and protein kinase C [19]. After binding to insulin, the CEACAM1 long cytoplasmic tail downregulates insulin receptor (IR) mitogenic signaling: IR phosphorylation of Tyr493 at the CEACAM1 cytoplasmic tail and recruitment of the adaptor protein Shc leads to Grb2/SOS sequestration, effectively reducing IR signaling (Figure 4) [79]. Similarly, CEACAM1 reduces epidermal growth factor receptor (EGFR) dependent cell proliferation by binding and sequestering Shc (Figure 4) [80]. The data suggest that CEACAM1 functions as a tumor suppressor in the early stages of tumorigenesis, as knockout or loss of CEACAM1 is associated with increased tumorigenic potential [81–84].

Immune cell suppression occurs after Src family kinase protein, lymphocyte-specific protein tyrosine kinase (LCK), phosphorylates the ITIMs of dimeric long tail CEACAM1, leading to recruitment of SHP2, which dephosphorylates the T cell receptor/CD3 complex and ZAP70, abrogating downstream signaling and activation of the T cell (Figure 4) [85]. CEACAM1 also downregulates natural killer cells and is involved in developing and differentiating a variety of myeloid-derived immune cells [5]. Knockout of CEACAM1 leads to the development of exhaustion-resistant and hyperinflammatory T cells, a phenotype mediated by CEACAM1-TIM-3 interactions [36]. CEACAM1 is a regulator of TIM-3, binding to TIM-3 through its N-terminal domain, forming a heterodimer, and facilitating the maturation and cell surface expression of TIM-3, which inhibits T cell activation and prevents the development of exhaustion-resistant and hyperinflammatory T cells [36]. CEACAM1 also plays a regulatory role in NK-cell-mediated cytolysis, evading NK cells by promoting intracellular retention of several NKG2D ligands [35]. Melanocytes normally do not express CEACAM1, but it was found that in melanoma cells increased expression of CEACAM1 and decreased NKG2D ligands, lead to decreased susceptibility to NK cell-mediated cytolysis [35].

Under apoptotic conditions in CT51 mouse colon carcinoma cells, CEACAM1-L is cleaved at the ^457^DQRD^460^ motif by caspase-3 within its long cytoplasmic domain, resulting in degradation of its 8 kDa cytoplasmic domain and formation of a truncated version of CEACAM1-L, the CEACAM1-LΔ461 mutant [86]. NIH 3T3 cells stably expressing mutant CEACAM1-LΔ461 show increased cell adhesion properties [86]. Cleavage of CEACAM1-L in apoptotic cells was blocked when a specific caspase-3 inhibitor was introduced, indicating CEACAM1-L is cleaved by caspase-3, but not caspase-7 or caspase-8 [86]. It remains unclear if CEACAM1-LΔ461 is tumor-promoting or tumor-suppressive. Crosslinking of CEACAM1 on the cell surface of tumor cells using monoclonal antibodies specific for CEACAM1 leads to apoptosis [84]. In a healthy colonic crypt mucosa, epithelial cells migrate towards the intestinal crypt lumen until they undergo apoptosis and shed into the gut lumen [84, 87]. Most colon tumors exhibit a marked reduction of apoptosis and decreased CEACAM1 [84]. In two cell lines of HT29 cells (colorectal adenocarcinoma cells), induction of CEA did not affect the low CEACAM1 cell line. However, it caused dose-dependent induction of apoptosis in the high CEACAM1 cell line [43], demonstrating that CEACAM5 is a mediator of CEACAM1-induced apoptosis. CEACAM1 expression is reduced in more than 85% of early colorectal adenomas and carcinomas [83]; a significant reduction is apparent in colonic aberrant crypt foci and hyperplastic polyps [83, 88]. Isolated Jurkat cells and HT29 cells displayed drastic improvement in apoptotic ability when CEACAM1 levels were high, indicating that loss of apoptosis is associated with decreased CEACAM1 expression in colorectal epithelial cells and lymphocytes [89].

Preserving the interactions between CEACAM1 and CEACAM5 might be critical in preventing colorectal adenocarcinomas. Interestingly, loss of CEACAM1 is associated with abnormal T cell function [41]. CEACAM1^−/−^ mice exhibit significantly altered CD8^+^ T cell activity in the gut mucosal tissue, with CD8^+^ T cells in CEACAM1^−/−^ mice expressing abundant CD29, yet less CD62L, enhancing secretion of proinflammatory cytokines IL-6, IL-17 and TNFα [41]. In addition, PD-1 and CTLA-4 are strongly upregulated on CD8^+^ T cells from CEACAM1^−/−^ mice infected with *Citrobacter rodentium*, but neither molecule is upregulated on CD8^+^ T cells from infected WT mice [41]. Differences in phenotype are even stronger under colitis and are likely responsible for the higher bacterial burden and defective intestinal barrier in CEACAM1^−/−^ mice exposed to *C. rodentium* induced colitis. Restoring CEACAM1 in T cells may prove critical in preventing inflammation and restoring apoptosis, as most colorectal tumors express decreased levels of CEACAM1 [83, 84].

Although its tumor suppressive properties are evident, the role of CEACAM1 in cancer is bimodal, with many similarities to the TGF-β signaling pathway; early effects being tumor suppressive, and more invasive cancers relying on it for malignant potential. For example, CEACAM1^−/−^ mice injected with highly metastatic MC38 colorectal cancer cells and B16F10 melanoma cells experience a reduction in the number and size of metastatic lesions compared to the WT littermate, with tumor cell proliferation decreasing by 2-fold and tumor cell survival decreasing by 3-fold, indicating that expression of CEACAM1 is associated with tumorigenesis in colorectal cancer [90]. CEACAM1 interacts with TNFα, playing a pathogenic role in cirrhosis-related hyperpermeability. CEACAM1 upregulates tumor necrosis factor-α (TNFα) in a positive-feedback loop [91]. The effect is increased apoptosis, disruption of tight junction protein-maintained barrier function, and decreased restitution capacity in causing colon adenocarcinomas with hyperpermeability and cirrhosis [86, 92]. Interrupting interactions between CEACAM1 and TNFα might prove effective in preventing cirrhosis-related hyperpermeability.

In hepatocellular carcinoma (HCC), CEACAM1 cytoplasmic expression is associated with poor differentiation, high recurrence rates, larger and greater number of tumors, vascular invasion, and satellite nodules in comparison with CEACAM1 membranous expression in HCC [32]. In the liver, loss of CEACAM1 in a murine model has been found to lead to inflammation and Non-Alcoholic Steatohepatitis (NASH), with reduced insulin clearance and altered metabolism [93]. Inflammation and NASH are risk factors for developing HCC in the CEACAM1 KO mice [93]. CEACAM1 has also been shown to be a binding partner to β2-spectrin, which is involved as a cofactor for SMAD3 in the TGF-β signaling pathway [43, 94, 95]. The expression of a long tail isoform of CEACAM1 leads to the nuclear localization of SMAD3 in HCC cell lines and leads to an increased invasive phenotype [43]. Rat HCC tumor cells transfected with CEACAM1^a^-4L displayed a significant decrease in tumor formation, progression, and burden compared to rat HCC cells transfected with CEACAM1^b^-4S and the negative control group, suggesting that CEACAM1 splice-switching therapy may inhibit HCC [96].

Exploring different isoforms of CEACAM1 (CEACAM1-L, CEACAM1-S, cytoplasmic CEACAM1, and membranous CEACAM1) is critical to better understanding the variable role of CEACAM1 in colorectal cancer, gastric cancer, and HCC, and can potentially explain the contrasting results in studies exploring CEACAM1 pathology in the gastrointestinal tract. A clinical study found that CEACAM1 was expressed in the sera of 24% (15/61) of normal patients, 66% (35/53) of patients with chronic pancreatitis, and 91% (74/81) of pancreatic cancer patients, indicating that CEACAM1 may be a viable biomarker for pancreatic cancer [97, 98]. In another study, CEACAM1 was shown to be upregulated in 69% of pancreatic carcinomas compared to 2% in normal patients, and patients with low serum CEACAM1 levels have significantly increased rates of overall survival [99]. Overall, CEACAM1 is downregulated in colorectal cancer, upregulated in gastric cancer, and directly correlated with decreased overall survival in HCC and gastric cancer [32, 82–84, 97, 98].

CEACAM5 is expressed in multiple epithelial malignancies, including gastric cancer, colorectal cancer, and pancreatic cancer, as well as in NSC lung cancer and melanoma [12, 100, 101]. In coordination with CEACAM1s ability to downregulate immune cells, CEACAM5 is a binding partner to CEACAM1 that can elicit this signaling, allowing for immune evasion by cancer cells [102]. Therapies targeting CEACAM5 in cancer are being developed to block this evading immune function [103, 104]. CEACAM5 has also been shown to be important in metastasis in colorectal cancer. In colorectal cancer, it was found to prevent anoikis by binding to DR5, leading to decreased activation of caspase 8 [105]. CEACAM5 also modulates the environment in the liver to create a space on the sinusoidal endothelial cells suitable for the adhesion and survival of metastatic cells by upregulating cytokine release and increasing protection against reactive oxygen species [106].

The TGF-β pathway plays an integral role in the suppression of early colorectal cancers, yet in advanced colorectal cancers, it plays a prominent tumor promoting role—in some ways similar to CEACAM1 function [107]. CEACAM5 binds to TGF-β type I receptor (TBR1) with decreased expression of SMAD3 targets, indicating that CEACAM5 can directly inhibit the tumor suppressive properties of TGF-β [108]. The same model showed that targeting CEA or its expression rescued TGF-β signaling. Interestingly, the expression of CEACAM5 and 6 is upregulated by SMAD3-mediated TGF-β signaling, suggesting a link between the metastatic properties of TGF-β in cancer and CEACAMs [109]. Decreased expression of the TGF-β pathway has also been inversely correlated with the expression of CEACAMs. Reduction in expression of TGF-β pathway members (TBR2, SMAD4, SPTBN1) has been shown to alter the colonic microbiome shifting towards a prevalence of bacteria associated with colorectal cancer [110]. The expression of CEACAM5 and CEACAM6 in colon cancer cell lines is increased in the presence of proinflammatory cytokine interleukin 6, supporting an important role in inflammation in colorectal cancer [111].

Similar to CEACAM5, CEACAM6 expression is significantly increased in malignancies [14, 15, 112]. In addition, CEACAM6 is a useful prognostic tool for cancer [15, 113, 114]. In a cohort of 115 lung adenocarcinoma patients, expression of CEACAM6 was associated with a five-year disease-free survival rate of 49.1%, as opposed to 74.2% for CEACAM6-negative patients [8]. In a study of gastric cancer patients, it was noted that an increased level of CEACAM6 DNA in the peripheral blood was significantly associated with a higher stage of the disease as well as lymph node metastasis [115]. Analysis of tumor specimens of gastric carcinoma patients using immunohistochemistry revealed increased CEACAM6 protein levels were associated with a higher stage, and that high CEACAM6 protein levels were associated with a shorter recurrence free survival [113]. Similarly, in colon cancer patients, higher levels of CEACAM6 expression was associated with higher tumor stage, and a shorter recurrence free survival [114].

CEACAM6 is important for the metastatic potential of cancers [114, 116, 117]. CEACAM6 was found to inhibit anoikis in the pancreatic ductal adenocarcinoma (PDA) line MiaPaca2, and inhibition of CEACAM6 with short interfering RNA (siRNA) led to decreased metastasis in a nude mouse orthotopic xenograft model [118]. The PI3K-AKT signaling pathway is involved in cell survival and silencing of CEACAM6 in the MiaPaca2 cell line led to decreased AKT phosphorylation [118]. Cross linking of CEACAM6 in BxPC3 cells using antibodies leads to resistance to anoikis by activating Src in a caveolin-1 dependent manner, leading to activation of focal adhesion kinase which then signals downstream to activate several cell proliferation and survival pathways [119]. In pancreatic cancer cell lines PANC-1 and CFPAC-1, CEACAM6 is involved in the expression of epithelial to mesenchymal transition-associated genes ZEB1 and ZEB2, thereby increasing the metastatic potential of cells [116]. Downregulation of CEACAM6 increased E-Cadherin levels in colon cancer cell lines, suggesting that CEACAM6 is a factor directly responsible for these cells’ invasive properties [114].

## MOUSE MODELS OF CEACAMS

The CEACAM1a null mice were initially used to provide insight into the role of CEACAM1 in insulin clearance [22, 96] and its role as a tumor suppressor in colon cancer [81], and more recently, in chronic viral infections [37] and immune regulation [36]. In CEACAM1 null mice, the absence of CEACAM1 led to an inability to clear insulin, leading to hyperinsulinemia followed by insulin resistance, obesity, and non-alcoholic steatohepatitis [120]. CEACAM1^−/−^ mice, WT mice, and CEACAM1-4S transgenic mouse overexpressing the mouse 4S isoform (4S Tg mice) have diverse microbiomes, and the 4S Tg mice exhibit increased *Gemella morbillorum*, *Lactobacillus murinus* and *Lactobacillus acidophilus*, suggesting that alteration of CEACAM1-4S expression can change the relative populations of certain microorganisms in the gut [31]. The CEABAC mouse model expresses CEACAM5, CEACAM6, and CEACAM7 in various tissues and almost matches the expression patterns in humans. This bacterial artificial chromosome does not include CEACAM4 and 8, and the model utilizes continued expression of endogenous CEACAM1. Nonetheless, in the CEABAC mice, a high fat and high sugar diet, termed a “Western diet,” modifies the composition of the gut microbiome and alters the environment in the intestines, leading to increased susceptibility to AIEC, supporting the idea that IBD is a multifactorial disease involving environmental and genetic factors [121]. This model has also been used to describe an approach to treating colitis with CAR-Treg cells specific to CEA [48]. In this model, CAR-Treg cells targeted towards CEA were administered to CEABAC mice in which colitis was induced by chemical methods such as dextran sodium sulfate ingestion, or by administering CEA specific CAR CD4^+^ effector T cells that would lead to inflammation. The mice treated with CAR Treg cells had reduced colitis morbidity in both models of colitis [48]. Tissue from CEABAC mice was used to analyze the effects of compounds that impede the binding of AIEC [122]. However, the limitations of this model are not to be understated. The interaction that usually exists between human CEACAM6 and human CEACAM1 does not exist in this model, as murine CEACAM1 is expressed, which impedes the ability to study the interactions of human CEACAM6 and human CEACAM1 and their potential immune effects [40]. Of note, human CEACAMs are heavily modified post-translationally and the machinery for doing so is not fully present in a mouse, leading to a complex problem to fix [20].

Other murine models have also been developed, notably a human CEACAM5 transgenic mouse [21], a human CEACAM1 transgenic mouse [61], and a dominant negative phosphorylation defective CEACAM1 mutant that is overexpressed in the liver (L-SACC mouse) [123]. The L-SACC mice proved their utility in early studies that investigated the role of CEACAM1 in the clearance of insulin and insulin receptors and its effects on metabolism and the liver [123, 124]. Studies involving the CEACAM5 transgenic mouse focused mainly on developing therapies that targeted the CEACAM5 protein in cancer [49, 125–127]. They were also used to demonstrate the ability of a CEACAM5 antibody to recognize liver metastasis in colorectal cancer [128]. The mouse models are outlined in Table 1.

**Table 1:** Mouse models involved in studying CEACAM biology

Gene(s) and organism of origin	Murine model	Model design	Disease relevance	Model phenotype	Refs
CEACAM1 Mouse	CEACAM1 KO CEACAM1 knockout (C57BL/6)	Disruption of CEACAM1 exon 1 and 2 with neomycin cassette	Liver cancer	By 3 mo, mice develop pericellular liver fibrosis By 5 mo, mice exhibit increased liver mass and hepatic triglyceride content. By 3–12 mo, mice show accelerated progression to hepatic steatosis. By 12–14 mo, mice show a higher number and significantly larger lipid vesicles. Increased susceptibility to NASH, portal inflammation, and lobular inflammation.	[137, 138]
CEACAM1 Mouse	CEACAM1 KO CEACAM1 knockout (C57BL/6)	Disruption of CEACAM1 exon 1 and 2 with neomycin cassette	Colon cancer	By 22 mo, mice do not spontaneously develop colon tumors	[81]
CEACAM1a Mouse	CEACAM1a KO CEACAM1 knockout (C57BL/6)	Disruption of CEACAM1a exon 1 and 2 with neomycin cassette	Hepatitis	By 3 wk, mice inoculated with 10^6^ PFU of Mouse hepatitis virus (MHV)-A59 were fully resistant to MHV-A59 infection by both intranasal and intracerebral routes	[22]
CEACAM1 Rat	Mutant CEACAM1 (L-SACC1) Liver Specific overexpression of dominant negative phosphorylation- defective S503A-CEACAM1 (C57BL/6 × FVB)	Insertion of rat CEACAM1 intron 1 between *Nde*I at nt 10 in exon 1 and *BamH*I at nt 313 in exon 2	Type 2 diabetes	By 3 mo, L-SACC1 mice exhibit insulin resistance	[124]
CEACAM1 Rat	Mutant CEACAM1 (L-SACC1) Liver Specific overexpression of dominant negative phosphorylation- defective S503A-CEACAM1 (C57BL/6 × FVB)	Insertion of rat CEACAM1 intron 1 between *Nde*I at nt 10 in exon 1 and *BamH*I at nt 313 in exon 2	Liver cancer	Mice exhibit increased hepatocyte proliferation, visceral obesity, and levels of circulating adipokines	[80]
CEACAM1 Human	h*CEACAM1* transgenic mouse (huTg) CEACAM1 heterozygosity (C57/BL6 × FVB/NJ)	BAC3 and BAC5 sites flanking human CEACAM1 gene with BAC2 covering a large region proximal to human *CEACAM1* on chromosome 19	Gonorrhea, meningitis	Mice display increased levels of Neisseria Opa52 positive bacteria bound to neutrophils.	[61]
CEACAM1 Human	h*CEACAM1* transgenic mouse (huTg) CEACAM1 heterozygosity (C57/BL6 × FVB/NJ)	BAC3 and BAC5 sites flanking hCEACAM1 gene with BAC2 covering a large region proximal to human *CEACAM1* on chromosome 19	Liver cancer	By 24 wk, mice have both increased susceptibility to liver cancer and increased intake of diacyl glycerides. By 25 mo, 17.6% of CEACAM −/− mice survived (compared to 83.3% of WT). By 25 mo, 38% of mice die from liver cancer (compared to 0% of WT).	[139]
CEACAM1-4L Human	h*CEACAM1-*4L transgenic mouse T cell-specific overexpression of the CEACAM-4L (C57BL/6 mice)	EcoRI sites flanking VACD2 cassette, cloned into pBS-SK(-), driven by hCD2 promoter	Cancer	Decreased T cell proliferation	[34]
CEACAM1 Human, Mouse	*hCEACAM1*^+/+^ × *msCeacam1^−/−^* msCEACAM1 knockout and hCEACAM1 expression (C57BL/6)	hCEACAM1 transgenic mice were back-crossed into the C57BL/6 background, and then crossed with mouse ceacam −/− mice to generate human CEACAM1 only expressing transgenic mice	Chronic viral infection	Mice exhibit increased resistance to lymphocytic choriomeningitis virus	[37]
CEACAM3, CEACAM5, CEACAM6, CEACAM7 Human	CEABAC10 Expression of hCEACAM3, hCEACAM5, hCEACAM6, and hCEACAM7	hCEABAC DNA was cloned into the pBeloBAC11 vector at the HindIII restriction site, and flanked by two *Not*I restriction sites.	IBD	By day 6, CEABAC10 mice have a 3.0-fold increase in intestinal permeability and disruption of mucosal integrity in a type 1 pili-dependent mechanism compared to WT mice	[140]
CEACAM3, CEACAM5, CEACAM6, CEACAM7 Human	CEABAC2 or CEABAC10 (dependent on transgene copy number)	hCEABAC DNA was cloned into the pBeloBAC11 vector at the HindIII restriction site, and flanked by two *Not*I restriction sites.	Colon cancer	By 20 wk post treatment, mice have more than a 2-fold increase in mean tumor load relative to their wild-type littermates	[141]
CEACAM3, CEACAM5, CEACAM6, CEACAM7 Human	CEABAC10 Expression of hCEACAM3, hCEACAM5, hCEACAM6, and hCEACAM7 (C57BL/6)	hCEABAC DNA was cloned into the pBeloBAC11 vector at the HindIII restriction site, and flanked by two *Not*I restriction sites.	IBD	Mice express no significant change in the intestinal microbiome	[121]
CEACAM1, CEACAM3, CEACAM6 Human	hCEACAMall Myeloid cell-specific expression of hCEACAM1, CEACAM3 and CEACAM6 (C57BL/6)	Cross-breeding between CEABAC10 mice and hCEACAM1 mice	Gastric cancer	Increased susceptibility to *Helicobacter Pylori* translocation of CagA into neutrophils	[142]

## PERSPECTIVE

CEACAM1, 5, and 6 have shown their importance in gastrointestinal pathologies as they are closely involved with immune regulation, tumorigenesis, tumor suppression, and pathogen binding. Interrupting the interactions of CEACAM family members seems to be an interesting avenue of research, as it may be a useful tool in cancer therapy [106], and regulating immune activity in the context of IBD [57]. However, much work has yet to be done in regard to the role of CEACAMs in cellular signaling and their effects on microbiome regulation.

Key questions that remain unanswered are whether CEACAMs are drivers or sensors or both of the gut microbiome and their signaling pathways/axes from the gut to the liver and brain in modulating disease and cancer. Some of these can be addressed through existing mouse models such as CEACAM1. However, for the most part, CEACAM5 and 6, which are expressed in humans but not rodents, these interactions could be addressed through new recent and technological advances, such as STORM (a super-resolution light microscopy method that uses fluorescent probes to separate overlapping regions of individual molecules so that the position of each molecule can be more clearly determined, allows for single-molecule imaging and creation of three-dimensional fluorescence images of cells and tissues), and Cryo-electron microscopy (CryoEM)- a technique that flash-freezes microscopic protein samples in a single-molecule-thick layer of vitreous ice, provides new insight into the structure and assembly of macromolecules [129, 130]. Similarly, organoids self-assembled *in vitro* three-dimensional structures that are primarily generated from primary tissues or stem cells, mimic the complex aspects of their organ counterparts [131]. Organoids simulating colorectal cancer, pancreatic cancer, the gut microbiome, and HCC could prove to be an important next step. In addition, liver organoids, expressing hepatocytes, endothelial cells, stellate cells, cholangiocytes, mesenchymal, and Kupffer cells, have been developed to explore fibrosis, NAFLD, hepatobiliary functions, and transplant capabilities [132–136]. Combining CRISPR and organoid technology, expressing different levels of CEACAMs in liver organoids, may contribute to clarifying the effect of obesity and diet on CEACAM expression and adhesion in HCC. AlphaFold, a software that predicts the 3D structure of a protein based on its genomic sequence will likely prove vital in exploring the lesser-known CEACAMs, including CEACAM4, CEACAM8, CEACAM16, CEACAM18, CEACAM19, CEACAM20, and CEACAM21, in pathology, oncology, the immune system, and the microbiome.
